# Supplementary material for: Predicting Transdermal Fentanyl Delivery Using Mechanistic Simulations for Tailored Therapy
Source: Front Pharmacol. 2020 Sep 29;11:585393. doi: 10.3389/fphar.2020.585393 (PMC7550783; doi:10.3389/fphar.2020.585393)
Supplement: Supplementary file 1 [file DataSheet_1.pdf]

# SUPPLEMENTARY MATERIAL

## 1 Derivation of conservation equation

The conservation equation was reformulated to another dependent variable to avoid numerical stability issues during calculation and to obtain a single equation that could be solved throughout the entire computational domain. In most studies (Table 1), in each material i (stratum corneum - sc, viable epidermis - ep, drug patch/reservoir - pt), the following conservation equations are solved for substance α:

$$\frac{\partial c_{pt}^{\alpha}}{\partial t} + \nabla \cdot \left( -D_{pt}^{\alpha} \nabla c_{pt}^{\alpha} \right) = 0$$

$$\frac{\partial c_{sc}^{\alpha}}{\partial t} + \nabla \cdot \left( -D_{sc}^{\alpha} \nabla c_{sc}^{\alpha} \right) = 0 \qquad (1)$$

$$\frac{\partial c_{vep}^{\alpha}}{\partial t} + \nabla \cdot \left( -D_{vep}^{\alpha} \nabla c_{vep}^{\alpha} \right) = 0$$

The concentrations in the different materials are linked via the partition coefficients by:

$$K_{pt/sc}^{\alpha} = \frac{c_{pt}^{\alpha}}{c_{sc}^{\alpha}} \qquad (2)$$

$$K_{sc/vep}^{\alpha} = \frac{c_{sc}^{\alpha}}{c_{vep}^{\alpha}} \qquad (3)$$

Additionally, flux continuity across the interfaces of different materials is assumed:

$$\left( -D_{pt}^{\alpha} \nabla c_{pt}^{\alpha} \right) \cdot \mathbf{n}_{pt-sc} = -\left( -D_{sc}^{\alpha} \nabla c_{sc}^{\alpha} \right) \cdot \mathbf{n}_{sc-pt}$$

$$\left( -D_{sc}^{\alpha} \nabla c_{sc}^{\alpha} \right) \cdot \mathbf{n}_{sc-vep} = -\left( -D_{vep}^{\alpha} \nabla c_{vep}^{\alpha} \right) \cdot \mathbf{n}_{vep-sc} \qquad (4)$$

In the conservation equations above, we substituted the dependent variable drug concertation ($c_i^{\alpha}$) with the dependent variable potential ($\psi_i^{\alpha}$) in each of these materials, based on following relations:

$$c_{pt}^{\alpha} = K_{pt}^{\alpha} \psi_{pt}^{\alpha} \qquad (5)$$

$$c_{sc}^{\alpha} = K_{sc}^{\alpha} \psi_{sc}^{\alpha} \qquad (6)$$

$$c_{vep}^{\alpha} = K_{vep}^{\alpha} \psi_{vep}^{\alpha} \qquad (7)$$

where $K_i^{\alpha}$ is termed the drug capacity in the material $i$ [-]. This capacity is defined to be related to the partition coefficient in the following way:

$$K_{pt/sc}^{\alpha} = \frac{K_{pt}^{\alpha}}{K_{sc}^{\alpha}} \qquad (8)$$

$$K_{sc/vep}^{\alpha} = \frac{K_{sc}^{\alpha}}{K_{vep}^{\alpha}} \qquad (9)$$

As such, when combining these equations, it follows that at the material interface:

$$\frac{K_{pt}^{\alpha}}{K_{sc}^{\alpha}} = \frac{c_{pt}^{\alpha}}{c_{sc}^{\alpha}} \quad \text{so} \quad \frac{K_{pt}^{\alpha}}{K_{sc}^{\alpha}} = \frac{K_{pt}^{\alpha}\psi_{pt}^{\alpha}}{K_{sc}^{\alpha}\psi_{sc}^{\alpha}} \quad \text{leading to} \quad \frac{\psi_{pt}^{\alpha}}{\psi_{sc}^{\alpha}} = 1 \qquad (10)$$

$$\frac{K_{sc}^{\alpha}}{K_{vep}^{\alpha}}=\frac{c_{sc}^{\alpha}}{c_{vep}^{\alpha}} \quad \text{so} \quad \frac{K_{sc}^{\alpha}}{K_{vep}^{\alpha}}=\frac{K_{sc}^{\alpha}\psi_{sc}^{\alpha}}{K_{vep}^{\alpha}\psi_{vep}^{\alpha}} \quad \text{leading to} \quad \frac{\psi_{sc}^{\alpha}}{\psi_{vep}^{\alpha}}=1 \tag{11}$$

Thus, we get $\psi_{pt}^{\alpha}=\psi_{sc}^{\alpha}=\psi_{vep}^{\alpha}=\psi^{\alpha}\left[kg/m^3\right]$. Hence, only one dependent variable ($\psi^{\alpha}$) needs to be solved for the entire domain, instead of several concentrations ($c_i^{\alpha}$) in each material $i$ separately. When substituting Eq. (15), (16), and (17) in Eq. (1), we get for each material i:

$$\frac{\partial K_i^{\alpha}\psi^{\alpha}}{\partial t}+\nabla\cdot\left(-D_i^{\alpha}\nabla K_i^{\alpha}\psi^{\alpha}\right)=0 \tag{12}$$

If we assume that $K_i^{\alpha}$ is constant within each material (so $\nabla K_i^{\alpha}=0$, except at the border between materials), we can write:

$$\nabla K_i^{\alpha}\psi^{\alpha}=K_i^{\alpha}\nabla\psi^{\alpha}+\underbrace{\psi^{\alpha}\nabla K_i^{\alpha}}_{=0}=K_i^{\alpha}\nabla\psi^{\alpha} \tag{13}$$

If we also assume that $K_i^{\alpha}$ is constant over time, Eq. (22) becomes:

$$K_i^{\alpha}\frac{\partial\psi^{\alpha}}{\partial t}+\nabla\cdot\left(-D_i^{\alpha}K_i^{\alpha}\nabla\psi^{\alpha}\right)=0 \tag{14}$$

This equation is valid over the entire computational domain, with one dependent variable ($\psi^{\alpha}$), which is continuous throughout all materials and over all interfaces. Only $K_i^{\alpha}$ and $D_i^{\alpha}$ are material-specific, by which no explicit continuity of fluxes needs to be imposed.

## 2 Validation simulations

The drug release and uptake model was validated with previous experimental data [21]. This process was performed by quantifying how accurately the drug uptake kinetics were predicted with our mechanistic model. The experiment considered fentanyl uptake of a cylindrical drug reservoir (radius 9 mm, thickness 50.8 µm) through a cylindrical skin sample (human cadaver epidermis, radius 9.25 mm, thickness 50.8 µm). Both components were fitted into a Franz diffusion cell and monitored at 33°C for 72 h. Several initial drug concentrations in the patch were evaluated. For the validation simulations, 60 and 80 kg $m^{-3}$ were considered. The flux in the experiments was determined over a specified time period of multiple hours by removing an aliquot of the receptor medium and analyzing the concentration via high-performance liquid chromatography (HPLC).

The conservation equations, boundary conditions, and initial conditions that were solved are similar to those detailed in section 2.1, but what follows is a brief description of the differences. A 3D cylindrical model of the setup was made. However, due to the very similar radius of the patch and the skin sample, the transport processes were quasi-one-dimensional. The small differences when comparing the 3D and 1D models for the validation study confirmed this fact (Figure 2). Thus, only the results for the 1D model are shown. The geometrical specifications and transport properties used in the simulations are indicated in Table 3. Note that the drug fluxes leaving the epidermis in experiments and simulations were scaled to the surface area at the patch-skin interface (surface 2, Figure 3), and not to that of the skin (which is slightly larger; surface 1).

## 3 Modeling assumptions

The impact of several modeling assumptions was evaluated. First, we evaluated whether a unidirectional (1D) transport model could be used as a simplification of the 3D transport problem. For this purpose, the base case for a 1D model ($L_{pt}$ = $L_{sk}$) was compared with the 3D model for the base case ($L_{sk} = L_{pt} + 20 \times (d_{sc} + d_{vep})$). This comparison implies quantifying whether the transport in the transverse direction impacts the solution for a standard patch size. A comparison of the uptake flow ($G_{bl,up}(t)$), the total amount that is taken up by the blood flow ($m_{bl,up}(t)$), and the maximal concentration in the skin ($c_{sc,max}(t)$), is shown in Figure S1, by quantifying the differences between both model types. The differences dropped below 1% within the first hour. This finding is not surprizing due to the large size of the patch: the ratio of the patch width to the epidermal thickness (stratum corneum and viable epidermis) was 471. For such very wide computational domains, a 1D approximation is clearly sufficiently accurate, because the edge effects do not affect the total released amount. Thus, in this study, the use of a 1D unidirectional transport model was justified. For smaller patch sizes, or in case the dermis would be included in the model, the use of 3D modeling should be re-evaluated, but this evaluation should be assessed on a case-by-case basis.

With regards to these findings, the impact of drug flow anisotropy in the stratum corneum was evaluated, because the diffusion coefficient in the transverse direction is much higher than the one in the longitudinal direction (Table 3). To this end, an isotropic versus anisotropic diffusion coefficient for the stratum corneum in the transverse direction was evaluated for a standard patch size. The base case (3D model, anisotropic diffusion coefficients) was compared with the case with isotropic diffusion coefficients. Here, the transverse diffusion coefficient was set equal to the longitudinal one, so much smaller than with the base case, namely $D_{sc}^{\alpha} = 7.2 \times 10^{-15}$ $m^2$ $s^{-1}$. The differences in uptake flow rate ($G_{bl,up}(t)$) were < 1%. Due to the large patch width, differences in transport in the transverse direction, which occur mainly at the patch edges, did not impact the total uptake flow rate.

Finally, the material properties used for the validation simulations (see [21]) differed from those used in the other simulations, which were taken from another study [26]. In the validation simulations, only one homogeneous epidermis layer was considered: the stratum corneum and the viable epidermis were embedded, which lumped their effect in one single diffusion and partition coefficient (Table 3). In our other simulations, the stratum corneum and the viable epidermis were discretely modeled, each with their diffusion and partition coefficient. We evaluated whether the utilized parameter set for the base case provided transport kinetics that were in the same range as the validated model parameter set. There was only a 20% difference in $m_{bl,up}(t)$ and $G_{bl,up}(t)$ between these two sources for material properties after the first 15 h. The difference in the total drug amount taken up at the end of the simulation was only 10%. Thus, both sets of material properties lead to similar transport kinetics. The main reason for this result is that the resistance to diffusion of both skin types for these two cases is very similar. This resistance R [s m$^{-1}$] for a skin built out of $n$ layers can be defined as:

$$R = \sum_{i=1}^{n} \frac{d_i}{D_i^\alpha} \tag{15}$$

where $d_i$ is the thickness of each layer [m]. The corresponding resistances for the diffusion coefficients of validation case and the base case were 0.733 x 10$^9$ and 2.084 x 10$^9$ s m$^{-1}$, respectively. Due to these similar diffusional resistances, the validation case can be considered representative of the other simulations. The partition coefficients also differ, by which the amount of drug stored in the skin will be different for both cases: the largest amount is stored for the material properties taken from the validation case.

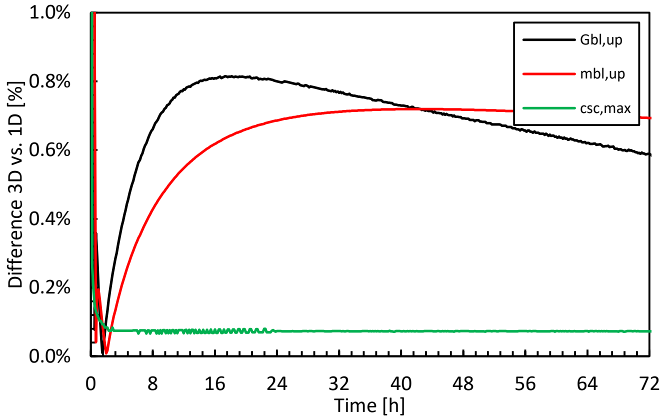


***Figure S1. Differences of 3D and 1D models concerning the uptake flow ($G_{bl,up}$), the total amount taken up by the blood flow ($m_{bl,up}$) and the maximal concentration in the skin ($c_{sc,max}$) as a function of time.***

## 4 Impact of the dermis layer

The impact of including the diffusion in the dermis on the diffusive drug uptake is illustrated by evaluating different dermis thicknesses, namely 100, 200, 400, 800, 1,600 and 3,200 µm. Only diffusion (and not blood flow) was modeled in the dermis. The uptake flux ($g_{bl,up}$) and the amount taken up by the blood flow ($m_{bl,up}$) are shown in Figure S2. Since no blood flow in the dermis was modeled, the time lag before the drug reaches the blood, i.e., the lower boundary of the computational domain increased significantly with increasing dermis thickness (Figure S2a). Furthermore, the drug amount taken up over 72 h decreased with increasing dermis thickness. Correspondingly, the stored drug amount increased (Figure S2b) and the peak in the uptake flux decreased.

From these results, the dermal blood flow should be included in the model when the dermis is explicitly modeled because the differences found in Figure S2a for the same drug reservoir were very large. It is not realistic that, if the size of the computational domain is increased, the predicted drug uptake by the patient decreases to such an extent. Hence, modeling the dermis without drug extraction by the blood flow likely underpredicts the drug uptake rate. Due to the large thickness and volume of the dermis, only modeling diffusive transport can also overpredict the stored drug amount and extensive transversal drug spreading in the dermis.

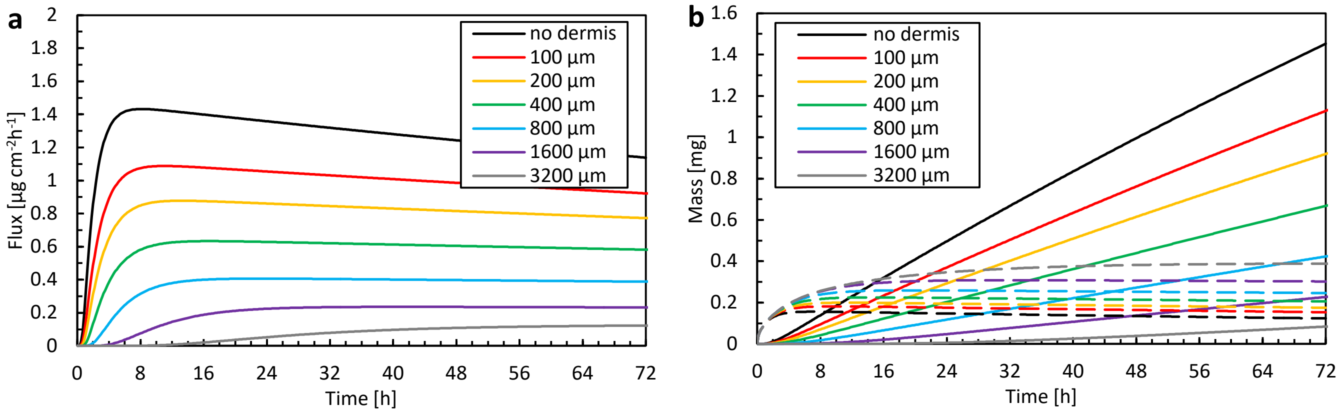


***Figure S2. The total amount that is taken up by uptake flux ($g_{bl,up}$) and the blood flow ($m_{bl,up}$) as a function of time for different thicknesses of the dermis.***
